# Randomized trial on the effects of an EMDR intervention on traumatic and obsessive symptoms during the COVID-19 quarantine: a psychometric study

**DOI:** 10.3389/fpsyt.2024.1369216

**Published:** 2024-06-26

**Authors:** Mario Miccoli, Andrea Poli

**Affiliations:** Department of Clinical and Experimental Medicine, University of Pisa, Pisa, Italy

**Keywords:** EMDR, COVID-19, psychological trauma, PTSD, OCD, disgust, guilt and shame, mental contamination

## Abstract

**Introduction:**

It has been suggested that the COVID-19 pandemic was a potentially traumatic occurrence that may have induced generalized anxiety and discomfort, particularly in susceptible populations like individuals with mental illnesses. The therapeutic approach known as eye movement desensitization and reprocessing (EMDR) has been shown to be successful in helping patients process traumatic events and restore wellbeing. Nevertheless, little is known about the precise processes through which EMDR fosters symptom recovery.

**Methods:**

In order to disentangle these issues, we conducted a randomized controlled trial (ClinicalTrials.gov Identifier NCT06110702) with 107 participants who were selected from university hospitals as a sample of investigation. Random assignments were applied to the participants in order to assign them to the experimental and control groups. The experimental group, but not the control group, underwent an 8-week EMDR intervention. Body perception, disgust, and emotions of guilt and shame, as well as mental contamination and posttraumatic and obsessive-compulsive symptoms, were investigated before and after the EMDR intervention.

**Results:**

The EMDR intervention was able to improve all of the variables investigated. Path analysis showed that body perception was able to predict both disgust and emotions of guilt and shame. Disgust was able to predict both mental contamination and obsessive-compulsive symptoms, while guilt and shame were able to predict post-traumatic symptoms.

**Conclusions:**

EMDR is an effective therapy for the treatment of post-traumatic and obsessive symptoms that acts through the promotion of improvement of the emotions of guilt/shame and disgust, respectively. Implications for clinical practice are examined.

**Clinical trial registration:**

https://www.clinicaltrials.gov, identifier NCT06110702.

## 1 Introduction

It has been suggested that the COVID-19 pandemic was a potentially traumatic occurrence (1, 2) that may have induced generalized anxiety and discomfort, particularly in susceptible populations like individuals with mental illnesses (3, 4). In particular, during the COVID-19 pandemic, an enduring life-threat feeling may be identified as a main traumatic trigger since it can lead everyone to be at risk to varying levels of contracting the illness, dying, infecting others, or losing loved ones. Distancing and avoiding company have a great impact on social life (5–8). Furthermore, it has been reported that the COVID-19 pandemic is likely to worsen pre-existing mental health conditions and may be involved in the emergence of new stress-related disorders for most people (9). The COVID-19 pandemic has been proposed to generate a higher and enduring proportion of traumatic symptoms than other potentially stressful events (10, 11).

Dramatic brain/body transformations (12) and a person’s altered relationship with self, others, and the world occur when experiencing multiple types of traumas, showing the need for body-oriented and sensorimotor therapies designed to remodel bodily self-aspects in the presence of traumatic conditions (13–20). In fact, mental health professionals and survivors of trauma really support the idea that trauma may emerge in the body, although the neurobiological bases of this manifestation are yet largely unknown (21). In particular, it was found that prior trauma exposure, body perception, and subsequent post-traumatic stress disorder (PTSD) played a main role in reactions to the COVID-19 pandemic (22). Hence, the assessment of body perception may inform the treatment of people overwhelmed by the COVID-19 pandemic. In terms of emotions, it has been shown that psychological trauma includes feelings of shame and guilt, which are known to be associated with PTSD symptoms (23–25). For example, COVID‐19 traumatic stress has been shown to predict PTSD symptoms after cumulative trauma (26) and may be able to trigger both guilt (i.e., “Am I infecting others?”) and shame (i.e., “Am I inferior or weak for being infected?”) (27). In addition, psychological distress, negative affect, positive affect, threatening COVID-19 illness perception, guilt, and shame were assessed and investigated in 351 participants who contracted COVID-19 in Israel. Interestingly, the authors found that threatening COVID-19 illness perception was linked to negative affect via guilt, and to psychological distress, negative affect, and positive affect via shame, suggesting that illness perception, shame, and guilt should be assessed and addressed by mental health professionals in people who contracted COVID-19 (28). Furthermore, in a sample of 72 Italian adults recruited in Italy, the traumatic symptom severity and negative emotions associated with COVID-19-related experiences were investigated as a primary outcome. The presence of traumatic symptoms was met by a total of 36%, and shame and fear activations predicted the scores of traumatic scales, suggesting the importance of shame in the maintenance of traumatic symptoms related to COVID-19 experiences (29). Interestingly, in the National Comorbidity Survey Replication, those with a current diagnosis of PTSD were 3.62 times more likely to have obsessive-compulsive disorder (OCD) (30–33), and estimates of comorbidity (from 19% to 31%) vary based on the principal diagnosis considered and on whether considering current or lifetime (current and past) diagnoses (34). In addition, it has been reported that between 30% and 82% of those diagnosed with OCD have a trauma history (35). In accordance with this, guilt and shame may also play a role in the development and maintenance of mental contamination (MC), the experience of dirtiness in the absence of a physical contaminant (36, 37). COVID‐19 traumatic stress has been shown to be able to exacerbate both MC and obsessive-compulsive symptoms (38) as assessed by the Dimensional Obsessive-Compulsive Scale (DOCS) (39). Specifically, MC was found to be predicted by the number of COVID-19 stressful events, compulsivity levels, and schizotypal symptoms, in particular in relation to magical thinking (40). In addition, disgust has been linked to MC in the prediction of PTSD symptoms (41–43) as well as in the prediction of fear of contamination and OCD symptoms (44–47). Considering that the COVID-19 pandemic was shown to have no effect on disgust sensitivity related to pathogens (i.e., the degree to which an individual is distressed by his/her experience of pathogen disgust), indicating that in a sample of adults from the United Kingdom disgust sensitivity is unchanged (48), it may be disgust propensity (i.e., the likelihood that an individual will experience a disgust reaction), in particular the pre-pandemic disgust propensity (49), a vulnerability factor for anxiety responses to the COVID-19 pandemic, particularly among individuals experiencing high stress (50). Hence, assessing disgust proneness and current stress may facilitate targeted anxiety intervention during the pandemic (51).

It has been highlighted that the COVID-19 pandemic has generated multiple excruciating and ethically difficult scenarios (e.g., not being able to tend to a sick or dying loved one) that may lead to subsequent guilt, shame, or moral injury. Therefore, trauma-informed guilt reduction therapy may help patients accurately appraise their role in a stressful event (such as those experienced during the COVID-19 pandemic) and find positive ways to express important values going forward (52). Eye movement desensitization and reprocessing (EMDR) is a psychotherapeutic approach that has demonstrated efficacy in the treatment of PTSD through several randomized controlled trials (53–57). The therapy follows the adaptive information processing (AIP) model (58–60), asserting that novel experiences are assimilated into memory networks via the brain’s innate information-processing machinery. EMDR is represented by an eight-phase protocol that can be guided by the therapist who can choose to deepen one, or more, of the eight phases before proceeding to another phase. Beyond PTSD, it has been reported that addictions, somatoform disorders, sexual dysfunction, eating disorders, adult personality disorders, mood disorders, response to extreme stress, anxiety disorders, performance anxiety, pain, neurodegenerative disorders, mental disorders of childhood and adolescence, sleep, and OCD are just some of the pathological conditions in which research has shown that EMDR is beneficial (61). In a sample of 57 victims of rape, early intervention with EMDR was not found to be more effective than watchful waiting in reducing PTSD symptoms, general psychopathology, depression, sexual dysfunction, and feelings of guilt and shame (62). However, several studies demonstrate the feasibility and efficacy of EMDR as an accessible therapeutic option for addressing mental health difficulties after the COVID-19 pandemic both online (63–66) and face-to-face (67). For example, a pilot study was carried out with 21 patients hospitalized for COVID-19 who were assessed for anxiety and depressive symptoms, intensity of distress, and levels of experienced fear (i.e., fear of the unknown) and were treated with EMDR therapy. After a four-session treatment, the EMDR therapy was shown to be effective in reducing all of the evaluated symptoms in all patients and promoted stabilization. All patients maintained improved psychological states for 1 week following the four sessions (67). Regarding online EMDR interventions, a sample of 38 patients with acute stress disorder were assessed for traumatic symptoms, as well as depression and anxiety, before and after the treatment and at the 1-month follow-up. After a seven-session online EMDR therapy, it was shown that the EMDR intervention was able to reduce anxiety by 30% and traumatic and depressive symptoms by 55% (64).

Here, using non-clinical experimental and control groups, our goal was to conduct a randomized controlled trial in order to evaluate the efficacy of an 8-week, face-to-face, EMDR treatment with participants who experienced a full COVID-19 pandemic-related quarantine during red zones of the second and third lockdowns in Italy. The following outcomes were hypothesized: a) body perception, disgust, guilt and shame, MC, obsessive-compulsive, and traumatic symptom scores in the experimental sample would ameliorate after the EMDR session, but not in the control sample; b) body perception would positively relate with disgust and emotions of guilt and shame.

## 2 Materials and methods

### 2.1 Trial design

As reported in the ClinicalTrials.gov Identifier NCT06110702 (https://clinicaltrials.gov/ct2/show/NCT06110702; accessed on April 14, 2024), our investigation is an interventional controlled trial with a randomized allocation. The main objective of the intervention method, which is based on a parallel assignment, is supportive care. The Declaration of Helsinki was followed in the conduct of the study.

### 2.2 Participants

The following were the eligibility criteria: participants had to be between the ages of 18 and 75 years, and both women and men were accepted as healthy volunteers. Inclusion criteria were as follows: participants aged 18 to 75 years, participants experienced a full COVID-19 pandemic-related quarantine during red zones of the second and third lockdowns in Italy, participants have reasonable comprehension of spoken and written Italian language, participants are willing to attend all intervention sessions, and participants are able to comprehend Italian to a sufficient degree. All of the participants included in the study were able to satisfy the Diagnostic and Statistical Manual of Mental Disorders, Fifth Edition (DSM-5) criteria for PTSD. A subgroup of the participants (28%) was able to satisfy DSM-5 criteria for OCD, as well. Exclusion criteria were as follows: concurrent participation in other intervention studies that may affect the effects of EMDR intervention in terms of process or outcome; subjects who previously underwent EMDR treatment in the past; subjects who previously experienced other non-EMDR treatments in the past; subjects with a previous history of psychiatric, or other medical illness, and with a previous history of use of psychotropic medication; refusal to give informed consent. Private psychotherapy centers in Prato, Florence, and Pisa hosted the EMDR intervention. A documented informed consent form was signed by each participant in the research.

### 2.3 Intervention

According to the Template for Intervention Description and Replication (TIDieR) guidelines (68), the “eye-Movement-Desensitize-reLabel (MDL) study” was conducted with two groups: an experimental group that participated in an EMDR intervention and a control group that went about their regular business as usual.

A total of eight experienced psychotherapists who are also certified EMDR supervisors or practitioners and experts in leading EMDR sessions with complex traumas conducted the EMDR sessions. An 8-week EMDR treatment with weekly 60-minute sessions was undertaken by the experimental group (69). A detailed description of the 8-week EMDR treatment is depicted in **Supplementary Table 1**.

### 2.4 Outcome measures

#### 2.4.1 Primary outcome measures

##### 2.4.1.1 Impact of Event Scale-Revised

The Impact of Event Scale-Revised (IES-R) (70) was used to assess post-traumatic symptomatology in compliance with the DSM fourth edition, text revision (DSM IV-TR) (71), validated and translated into Italian (72). Three subscales evaluating intrusion, avoidance, and hyperactivation are part of the 22-item IES-R instrument. A 5-point Likert scale, ranging from 0 (“*not at all*”) to 4 (“*a lot*”) referring to the past 7 days, was used to assess the participants’ degree of post-traumatic symptoms. The Italian translation of IES-R has shown satisfactory internal validity (72). In our study, IES-R showed a Cronbach’s α = 0.931.

##### 2.4.1.2 Dimensional Obsessive-Compulsive Scale

The DOCS (39) is a 20-item scale that assesses the main OCD symptom dimensions: contamination obsessions as well as washing and cleaning compulsions, obsessions about responsibility for harm and checking compulsions, repugnant obsessive thoughts and mental compulsive rituals or other covert neutralizing strategies, and obsessions about order and symmetry and ordering or arranging compulsions. Items evaluate five severity factors related to the previous month within each symptom dimension, and ratings can vary from 0 (no symptoms) to 4 (severe symptoms). The Italian version of the DOCS (73) replicated the four-factor structure of the original version. In our study, Cronbach’s α = 0.944.

##### 2.4.1.3 Body Perception Questionnaire-22 

The Body Perception Questionnaire (BPQ) (74) was initially developed by Porges (75) and later improved by Cabrera et al. (76) and Poli et al. (74) as a self-report test of body awareness and autonomic reactivity. In our study, the 22-item Italian version was used (74). Participants were asked to rate the frequency with which they feel aware of physical sensations [body awareness subscale (BOA), for example, “watering or tearing of my eyes”], as well as the frequency with which they experience supradiaphragmatic reactivity [supradiaphragmatic subscale (SUP), for example, “When I am eating, I have difficulty talking”] and subdiaphragmatic reactivity [subdiaphragmatic and body awareness subscale (BOA/SUB), for example, “After eating I have digestive problems”] on a 3-point scale (from 1 = *never* to 3 = *often*). In our study, BPQ-22 showed a Cronbach’s α = 0.888.

##### 2.4.1.4 Guilt and Shame Proneness Scale 

The Guilt-Negative-Behavior-Evaluation (NBE), Guilt-Repair, Shame-Negative-Self-Evaluation (NSE), and Shame-Withdraw subscales compose the 16 total items of the Guilt and Shame Proneness Scale (GASP) (77). Every item explains a circumstance that may cause guilt or shame, or a way to deal with a situation that could cause guilt or shame. Individuals were requested to use a 7-point rating system, with 1 indicating “*very unlikely*” and 7 indicating “*very likely*”, to describe how likely they are to feel the emotion or behave in the way outlined in the scenario. In this study, we used the Italian version validated by Poli et al. (submitted). Cronbach’s α for the GASP scale was 0.875.

##### 2.4.1.5 Three Domains of Disgust Scale 

This 21-item self-report scale (78) investigates disgust propensity on three subscales: pathogen disgust, sexual disgust, and moral disgust. Participants were asked to rate each item on a 6-point Likert scale from 0 (“*not at all*”) to 7 (“*extremely disgusting*”). The original version of the scale showed a trifactorial structure in different samples and good psychometric properties. In our study, we used the Italian version validated by Poli et al. (44). In our study, the Three Domains of Disgust Scale (TDDS) showed a Cronbach’s α = 0.887.

##### 2.4.1.6 Vancouver Obsessional Compulsive Inventory-Mental Contamination scale 

This 20-item scale (79) assesses issues related to MC. A 5-point rating system, ranging from 0 (meaning “*not at all*”) to 4 (meaning “*very much*”), is used by participants to score each item. Twenty-seven items comprised the original Vancouver Obsessional Compulsive Inventory-Mental Contamination scale (VOCI-MC) (80). The revised version was reduced to 20 items and showed sound psychometric properties (79). The Italian version of the scale showed a one-factor structure, good internal consistency, test–retest reliability, and construct validity (81). In our study, VOCI-MC showed a Cronbach’s α = 0.933.

#### 2.4.2 Secondary outcome measures

##### 2.4.2.1 Depression Anxiety Stress Scale-21 

The Depression Anxiety Stress Scale (DASS) (82) is a self-report questionnaire that enumerates negative emotional symptoms and comprises 21 items (83, 84). It measures stress, anxiety, and depression with three subscales. On a scale of 1 (indicating “*Did not apply to me at all*”) to 4 (indicating “*Applied to me most of the time*”), participants rated how often they had encountered a certain symptom over the previous week. Sound psychometric properties were shown by the original DASS-21, and its Italian translation (85) replicated the three-factor structure of the original version and has shown adequate internal consistency, test–retest reliability, and construct validity. In the current study, DASS-21 showed a Cronbach’s α = 0.950.

### 2.5 Sample size

We assumed a minimum sample size of 12 per group in order to execute our randomized controlled trial. Among consecutive patients admitted to the university hospital in Pisa for the psychological consequences attributed to the COVID-19 pandemic, 215 individuals were evaluated for eligibility. A total of 108 individuals who were screened for eligibility were not included in the study. Twenty-six individuals did not meet the inclusion criteria, while 82 declined to be enrolled in the intervention. The study enrolled a total of 107 subjects for randomized assignment.

### 2.6 Randomization

Individuals were randomly allocated in a 1:1 ratio to either the experimental group (EMDR) or the control group (everyday usual activities) using a computer-generated basic randomization sequence. After the baseline evaluation, randomization was carried out by a statistician who was not otherwise associated with the research and did not interact with the subjects involved in the research. The allocation was kept secret from the outcome assessors, and subjects were instructed not to disclose which group they were assigned to. The psychologists who conducted the intervention differed from those who evaluated the outcomes.

### 2.7 Statistical analyses

SigmaPlot^®^ 14 (Systat Software, Chicago, IL, USA), AMOS^®^ 27 (Analysis of MOmentum Structures; IBM Corp., Armonk, NY, USA), Weka 3.8.6 data mining software (86, 87), and SPSS^®^ 27 (IBM Corp., Armonk, NY, USA) were used for all statistical analyses. In order to confirm that the distributions were not normal, the Shapiro–Wilk test was carried out (88, 89). The Mann–Whitney rank sum test (MWRST) was used to verify that the ages between the control and EMDR groups were not statistically different, and in order to assess gender frequency, the χ^2^ test with Yates’s correction was used since the total number of events was between 40 and 200. For comparisons related to ordinal primary and secondary outcome variables, between and within groups, before and after treatment, Conover’s *post-hoc* group rank sum comparisons were performed after a two-way analysis of variance on ranks (tw-ANOVA) in order to compare the results against a control group. Hierarchical regression analyses were used to determine which models were best at predicting the DOCS and IES-R scales. The variance inflation factor (VIF) and the condition number (K(A) = ‖A‖ ‖A^−1^‖), which measures how sensitive the parameter estimates are to little changes in the data matrix (90, 91), were also calculated to account for multicollinearity. For the DOCS and IES-R scales (measures of obsessive and post-traumatic symptoms, respectively) to be predicted as a criterion, the model showing adjusted *R* ^2^ was considered.

In order to explore and confirm a possible path model, we employed AMOS^®^ 27.0. The *p*-values reported were two-tailed, and a *p*-value <0.05 was considered significant. Before performing path analysis, we analyzed the relationships between the variables. The absolute fit indices that we utilized in this study were χ^2^ and the root mean square error of approximation (RMSEA); the Tucker–Lewis index (TLI) and the comparative fit index (CFI) were the incremental fit indices that we employed in this study. RMSEA levels of 0.06 or below, together with CFI and TLI values of 0.90 or higher, were regarded as “good fit” results. A χ^2^ value that is closer to zero indicates a better fit. Model fit study did not recommend χ^2^ as a model fit criterion since it depends on the sample size used (92). Thus, we did not use it as a fit statistic and just reported in this research. The model fit criteria that we used were as follows (93): in terms of TLI and CFI, values of ≥0.90 and ≥0.95 respectively indicated acceptable and excellent fits; in terms of RMSEA, values of ≤0.08 and <0.06 respectively indicated excellent and acceptable fits. We also reported its 90% confidence interval (CI).

In order to assess the efficacy of EMDR therapy, machine learning models were built using the predictors BPQ-22, GASP, TDDS, VOCI-MC, DOCS, and IES-R to predict the EMDR treatment group. The *k*-fold cross-validation method was used. The *k* = 10 technique was employed, wherein *k* was set at 10, a number that was determined by testing to retain a low bias.

## 3 Results

### 3.1 Group comparisons

**Figure 1:**
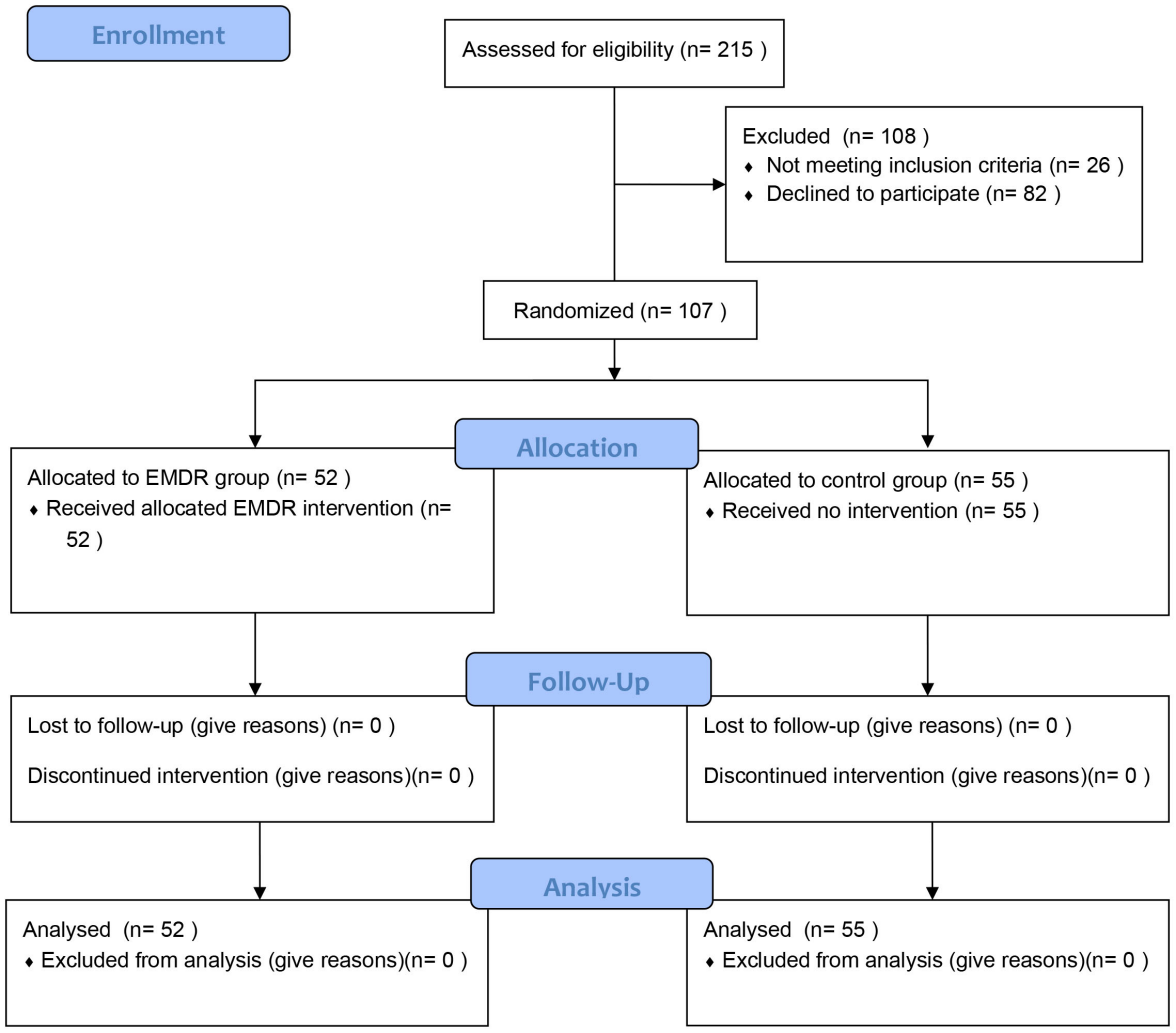
CONSORT flow diagram of the study.

Our research comprised 107 individuals (76 women, 71.03%; mean age = 44.03, SD = 11.13), as shown in the CONSORT flow diagram (94) (**Figure 1**). Fifty-five participants (37 women, 67.27%) were allocated to the control group, and 52 participants (39 women, 75%) were allocated to the EMDR group.

As a first step, we compared gender frequency and age between control and EMDR samples to evaluate gender and age homogeneity among groups. χ^2^ test revealed that gender frequency was not significantly different (p = 0.414) between the two groups, while MWRST showed that age was not significantly different (p = 0.719), as well. Thus, the control and EMDR groups were homogeneous regarding gender and age. Therefore, we examined participant scores in relation to the research measures both within and within the two groups (i.e., before and after EMDR treatment, respectively).

In relation to the pre-treatment baseline scores, tw-ANOVA with Conover’s *post-hoc* test revealed that secondary outcome measure DASS-21 scores (**Supplementary Tables 2**, **3**) were not statistically different between the EMDR and control groups (Control-Pre vs. EMDR-Pre: *p* = 0.803), while tw-ANOVA with Conover’s *post-hoc* test revealed that, after the EMDR treatment, EMDR samples showed scores that were statistically different (Control-Post vs. EMDR-Post: *p* < 0.001; EMDR-Pre vs. EMDR-Post: *p* < 0.001) with respect to control group (Control-Pre vs. Control-Post: *p* = 0.638). Most importantly, comparing post-intervention primary outcome measures, tw-ANOVA with Conover’s *post-hoc* test revealed that EMDR intervention was able to significantly ameliorate the scores of all the measures considered in the study. BPQ-22 scores were not statistically different between the EMDR and control groups (Control-Pre vs. EMDR-Pre: *p* = 0.215), while, after the EMDR treatment, EMDR samples showed scores that were statistically different (Control-Post vs. EMDR-Post: *p* < 0.001; EMDR-Pre vs. EMDR-Post: *p* < 0.001) with respect to the control group (Control-Pre vs. Control-Post: *p* = 0.484). GASP scores were not statistically different between the EMDR and control groups (Control-Pre vs. EMDR-Pre: *p* = 0.719), while, after the EMDR treatment, EMDR samples showed scores that were statistically different (Control-Post vs. EMDR-Post: *p* < 0.001; EMDR-Pre vs. EMDR-Post: *p* < 0.001) with respect to the control group (Control-Pre vs. Control-Post: *p* = 0.136). TDDS scores were not statistically different between the EMDR and control groups (Control-Pre vs. EMDR-Pre: *p* = 0.726), while, after the EMDR treatment, EMDR samples showed scores that were statistically different (Control-Post vs. EMDR-Post: *p* < 0.001; EMDR-Pre vs. EMDR-Post: *p* < 0.001) with respect to the control group (Control-Pre vs. Control-Post: *p* = 0.889). VOCI-MC scores were not significantly different between the control and EMDR groups (Control-Pre vs. EMDR-Pre: *p* = 0.276), while, after the EMDR intervention, EMDR samples showed scores that were significantly different (Control-Post vs. EMDR-Post: *p* < 0.001; EMDR-Pre vs. EMDR-Post: *p* < 0.001) with respect to control group (Control-Pre vs. Control-Post: *p* = 0.726). DOCS scores were not statistically different between the EMDR and control groups (Control-Pre vs. EMDR-Pre: *p* = 0.516), while, after the EMDR treatment, EMDR samples showed scores that were statistically different (Control-Post vs. EMDR-Post: *p* < 0.001; EMDR-Pre vs. EMDR-Post: *p* < 0.001) with respect to the control group (Control-Pre vs. Control-Post: *p* = 0.142). Finally, IES-R scores were not statistically different between the EMDR and control groups (Control-Pre vs. EMDR-Pre: *p* = 0.617), while, after the EMDR treatment, EMDR samples showed scores that were statistically different (Control-Post vs. EMDR-Post: *p* < 0.001; EMDR-Pre vs. EMDR-Post: *p* < 0.001) with respect to the control group (Control-Pre vs. Control-Post: *p* = 0.881) (**Tables 1**, **2**).

**Table 1:** Group comparisons among the study measures between EMDR (n = 52) and control (55) samples assessed pre-intervention and post-intervention.

Variable	Control-Pre	EMDR-Pre	*p*	Control-Post	EMDR-Post	*p*
1. BPQ-22	33.20 (8.02)	31 (7.39)	0.215	31.71 (7.64)	22.63 (5.76)	<0.001
	30 [10]	32 [12]		30 [9]	21.5 [8.75]	
2. GASP	62.36 (15.72)	61.26 (16.58)	0.719	67.13 (10.53)	49.65 (8.85)	<0.001
	65 [18]	63.5 [21.5]		68 [13]	50 [16]	
3. TDDS	71.64 (21.04)	68.94 (22.51)	0.726	70.31 (21.13)	36.60 (14.95)	<0.001
	72 [23.75]	72 [29.25]		71 [27.25]	40 [17.25]	
4. VOCI-MC	8.69 (11.19)	10.31 (12.26)	0.276	8.60 (11.99)	2.81 (2.47)	<0.001
	5.5 [7.75]	5 [17]		5.5 [9.75]	3 [3.75]	
5. DOCS	17.65 (13.16)	19.50 (15.42)	0.516	16.11 (14.35)	7.42 (6.12)	<0.001
	15 [18.25]	15 [21.75]		11 [18.75]	6 [9]	
6. IES-R	20.53 (16.41)	19.96 (14.83)	0.617	21.58 (14.26)	9.38 (5.92)	<0.001
	19.5 [25.5]	18.5 [20.75]		19.5 [23]	9.5 [9]	

p = p-value resulting from Conover’s post-hoc test from two-way analysis of variance on ranks for rows 1–6: 1. Body Perception Questionnaire-22; 2. Guilt and Shame Proneness scale; 3. Three Domains of Disgust Scale; 4. Vancouver Obsessional Compulsive Inventory-Mental Contamination; 5. Dimensional Obsessive Compulsive Scale; 6. Impact of Event Scale-Revised. Mean and standard deviation (in brackets), and median and interquartile range (in square brackets) are shown for rows 1—6.

**Table 2:** Group comparisons among the study measures within EMDR (n = 52) and control (55) samples assessed pre-intervention and post-intervention.

Variable	Control-Pre	Control-Post	*p*	EMDR-Pre	EMDR-Post	*p*
1. BPQ-22	33.20 (8.02)	31.71 (7.64)	0.484	31 (7.39)	22.63 (5.76)	<0.001
	30 [10]	30 [9]		32 [12]	21.5 [8.75]	
2. GASP	62.36 (15.72)	67.13 (10.53)	0.136	61.26 (16.58)	49.65 (8.85)	<0.001
	65 [18]	68 [13]		63.5 [21.5]	50 [16]	
3. TDDS	71.64 (21.04)	70.31 (21.13)	0.889	68.94 (22.51)	36.60 (14.95)	<0.001
	72 [23.75]	71 [27.25]		72 [29.25]	40 [17.25]	
4. VOCI-MC	8.69 (11.19)	8.60 (11.99)	0.726	10.31 (12.26)	2.81 (2.47)	<0.001
	5.5 [7.75]	5.5 [9.75]		5 [17]	3 [3.75]	
5. DOCS	17.65 (13.16)	16.11 (14.35)	0.142	19.50 (15.42)	7.42 (6.12)	<0.001
	15 [18.25]	11 [18.75]		15 [21.75]	6 [9]	
6. IES-R	20.53 (16.41)	21.58 (14.26)	0.881	19.96 (14.83)	9.38 (5.92)	<0.001
	19.5 [25.5]	19.5 [23]		18.5 [20.75]	9.5 [9]	

p = p-value resulting from Conover’s post-hoc test from two-way analysis of variance on ranks for rows 1–6; 1. Body Perception Questionnaire-22; 2. Guilt and Shame Proneness scale; 3. Three Domains of Disgust Scale; 4. Vancouver Obsessional Compulsive Inventory-Mental Contamination; 5. Dimensional Obsessive Compulsive Scale; 6. Impact of Event Scale-Revised. Mean and standard deviation (in brackets), and median and interquartile range (in square brackets) are shown for rows 1–6.

### 3.2 Hierarchical regressions

The use of linear regression analyses does not require that any of the observed variables be normal; nonetheless, in order to obtain a valid result by hypothesis testing, models should result in errors that should be normally distributed (95, 96). Thus, we conducted hierarchical regression analyses and took into account all the interval variables that had a significant impact after EMDR psychotherapy in order to determine the best models predicting DOCS and IES-R scores. The VIF was calculated for each predictor and was found within the range (1.05–1.28), which is in line with a lack of multicollinearity (97). Regarding the condition number, values greater than 30 are regarded as an index of multicollinearity (90, 91). In our study, the condition number was 14.874. Results of the hierarchical regression analysis predicting DOCS and IES-R for the post-intervention EMDR group are shown in **Supplementary Table 4**.

First, we evaluated which of the scales were able to predict DOCS. DASS-21 (β = −0.098, p = 0.566), BPQ-22 (β = 0.128, p = 0.479), GASP (β = −0.004, p = 0.978), TDDS (β = −0.007, p = 0.964), and IES-R (β = 0.112, p = 0.447) scales were not significant predictors of DOCS. VOCI-MC was the unique scale to be able to predict DOCS scores at the significance threshold (β = 0.383, p = 0.026). Subsequently, we evaluated which of the scales were able to predict IES-R. DASS-21 (β = −0.151, p = 0.383), BPQ-22 (β = 0.259, p = 0.155), GASP (β = −0.159, p = 0.313), TDDS (β = −0.058, p = 0.690), and DOCS (β = 0.115, p = 0.447) scales were not statistically different predictors of IES-R. VOCI-MC scores were confirmed as unique to be able to predict IES-R scores at a significance threshold (β = 0.367, p = 0.037).

### 3.3 Path analysis

AMOS^®^ 27 was used to examine path analytical models for the EMDR group post-intervention and to evaluate possible specific associations among the considered variables (98). Path analysis may allow researchers to compare various models to examine which one best fits the data and to analyze models that are more elaborated and realistic than multiple regression (99). Path analysis is a specific kind of structural equation modeling (SEM), which is a derivation of general linear models (GLMs). A momentum structural connection between variables of interest is the basis for the second generation of data analysis methods or GLM. Software such as AMOS^®^ 27 (100) may be used for SEM.

**Figure 2:**
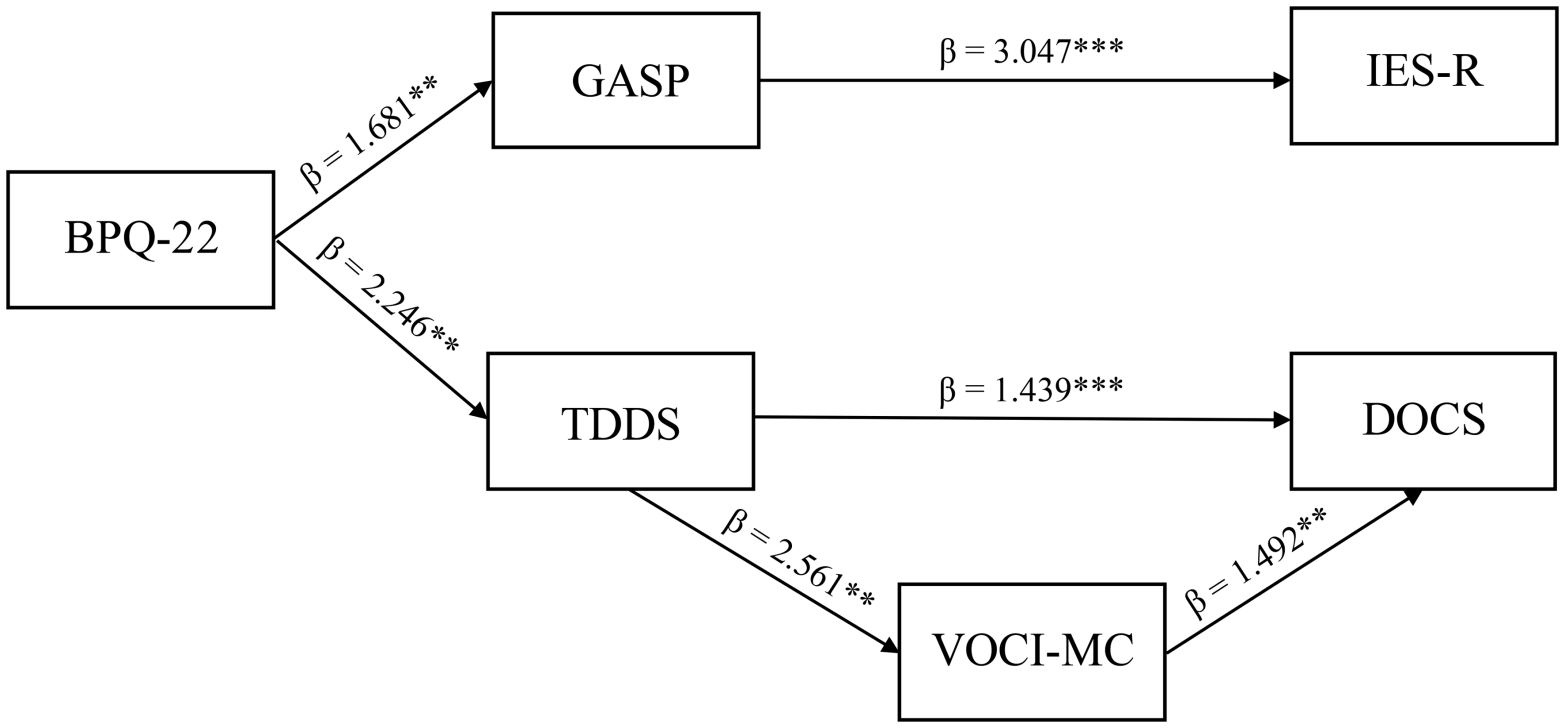
Path analytic model of the study. ** *p* < 0.01, *** *p* < 0.001

Analyzing the post-treatment EMDR sample, the BPQ-22 scale was found to act as a predictor of both GASP (β = 1.681, p = 0.006, SE = 0.069) and TDDS (β = 2.246, p = 0.002, SE = 0.058). In turn, GASP was found to act as a unique predictor of IES-R (β = 3.047, p = 0.001, SE = 0.066), while TDDS was found to predict DOCS scores (β = 1.439, p = 0.005, SE = 0.122). However, the effects of the TDDS on the DOCS scores were partially mediated by VOCI-MC (TDDS effects on VOCI-MC: β = 2.561, p = 0.003, SE = 0.058; VOCI-MC effects on DOCS: β = 1.492, p = 0.009, SE = 0.141). This model was found to achieve the highest fit among all of the models evaluated through the combinations of the considered variables (χ^2^ (7) = 3.942, p = 0.91, CFI = 0.987, TLI = 0.988, RMSEA = 0.028 [0.031; 0.037]) (**Figure 2**).

### 3.4 Machine learning analysis

We carried out an ML analysis to confirm the prediction of accurate categorization for the EMDR treatment group by the investigated factors. This allowed us to confront path analysis and ML algorithms (101) and to generate a hierarchical classification of the considered factors. In **Table 3**, ML classifier results are shown. The following five classifiers were compared: Hoeffding Tree [no. correct classification: 50/52; accuracy: 96.15%; area under receiver operating characteristic (ROC) curve: 0.917], Random Tree (no. correct classification: 51/52; accuracy: 98.08%; area under ROC curve: 0.949), Logistic Regression (no. correct classification: 50/52; accuracy: 96.15%; area under ROC curve: 0.901), and Naïve Bayes (no. correct classification: 51/52; accuracy: 98.18%; area under ROC curve: 0.954), Simple Logistics (no. correct classification: 50/52; accuracy: 96.15%; area under ROC curve: 0.923), and Logistic Regression (no. correct classification: 50/52; accuracy: 96.15%; area under ROC curve: 0.901).

**Table 3:** Accuracy, area under ROC curve, and number of correct classifications resulting from different machine learning classifiers, using 10-fold cross-validation, for EMDR group (n = 52) sample assessed post-intervention.

Machine learning classifier	Accuracy (%)	Area under ROC curve	Correct classification (no. correct/total)
1. Naïve Bayes	98.08	0.954	51/52
2. Simple Logistics	96.15	0.923	50/52
3. Logistic Regression	96.15	0.901	50/52
4. Hoeffding Tree	96.15	0.917	50/52
5. Random Tree	98.08	0.949	51/52

ROC curve, receiver operating characteristic curve.

## 4 Discussion

Our research highlights that EMDR treatment may ameliorate BPQ-22, TDDS, GASP, VOCI-MC, DOCS, and IES-R scales with respect to the control group that underwent no intervention following routine daily living. Research indicates that EMDR requires paying attention to the selected worst image related to the negative event, along with its associated bodily sensations, emotions, and physical sensations, which can facilitate a resumption of the learning process. This new form of learning may allow the event to be reprocessed in a way that is adaptive and no longer dysfunctional (102). As a matter of fact, once memory is retrieved, it may become labile again, which fosters its susceptibility to modification during a possible process of human memory reconsolidation (103, 104). Paying attention to the positive features after negative recall (like during the identification of positive cognition during the EMDR protocol) may lead to increased levels of positive emotion and modifications in memory content during recollection 1 week later, remaining even after 2 months. This evidence highlights the fact that in order to update maladaptive memories, a positive emotion-focused strategy is needed (105). Hence, the reduction of bodily discomfort and, concurrently, of a subjective unit of distress may be a prerequisite in order to obtain the identification and the installation of a positive cognition (106). After EMDR treatment, we found a significant amelioration in terms of perception of bodily discomfort, and this factor may play a pivotal role in order to achieve a remission of symptoms. The reduction of bodily discomfort, as measured by BPQ-22, may represent the starting point to achieve the reduction of guilt, shame, and disgust, as well as the installation of positive cognition.

Disgust is an understudied emotion; however, evidence suggests that it may play an important role in the development and maintenance of PTSD and, in many cases, may persist after the end of treatments (107). In a sample of 72 women with a history of sexual victimization, results demonstrated that while anxiety and disgust declined at similar rates across exposure trials, even after taking into account the severity of PTSD symptoms, ratings of disgust were higher at the beginning of exposure with respect to ratings of anxiety. In addition, for participants showing a significant reduction in anxiety, disgust modifications were able to significantly predict improvements in script-elicited PTSD symptoms throughout the period of exposure (108). Very recently, it has been shown that in a sample of 155 patients with a primary diagnosis of PTSD, 12 biweekly EMDR sessions were able to produce a significant improvement in disgust levels as well as PTSD symptoms measured by IES-R (109). Conversely, in our study, disgust was not found to play a role in the development and maintenance of PTSD symptoms. Possibly, the fact that we concurrently measured and examined OCD and PTSD-related symptoms may explain our results. In fact, it has been shown that a complex interplay exists related to the coexistence of PTSD and OCD. OCD and traumatic histories have significant overlap, and trauma should be considered when treating an individual with OCD (35). Accordingly, it has been shown that the coexistence of PTSD in OCD exacerbates obsessive-compulsive symptoms and increases the burden of anxiety (110). Hence, OCD symptoms may emerge to cope with the underlying trauma and PTSD. Therefore, when PTSD and OCD symptoms are assessed together, disgust may preferentially serve as a development factor for OCD symptoms in order to manage the underlying trauma and PTSD.

Regarding emotions of guilt and shame in a randomized controlled trial including 57 victims of rape, EMDR treatment has been shown to be beneficial in lowering symptoms of PTSD, emotions of shame and guilt, dysfunctional sexual behavior, depression, and general psychopathology (62). In addition, in the aforementioned study investigating a sample of 155 patients with a primary diagnosis of PTSD, 12 biweekly EMDR sessions were also able to generate a significant improvement in guilt and shame levels, as well (109). Our results are in line with these findings. Furthermore, using a path analytic model, we were able to reveal that guilt and shame levels may be direct predictors of PTSD symptoms, and in particular, reductions in guilt and shame levels may promote a direct amelioration of PTSD symptoms.

In terms of obsessive-compulsive symptoms, in a sample of 90 OCD patients who were randomly assigned to a 12-week EMDR treatment or citalopram group, it was reported that EMDR was found to be more beneficial than citalopram in ameliorating OCD symptoms (111). More recently, in a sample of 55 OCD patients who were randomly assigned to an EMDR treatment or cognitive-behavioral therapy (CBT) group, it was shown that both EMDR and CBT treatments were effective at reducing OCD symptoms and that EMDR and CBT showed analogous completion rates and clinical outcomes (112). The effectiveness of EMDR therapy in treating PTSD has undergone the scrutiny of several meta-analyses, and this led to the final recognition by the World Health Organization as a psychotherapy of choice in the treatment of PTSD in children, teenagers, and adults (113–119). In accordance with these results, our findings show that the EMDR intervention was able to improve both OCD and PTSD symptoms that were assessed simultaneously. Using a path analytic model, we were also able to hypothesize possible bodily and affective variables on which EMDR may intervene in order to achieve PTSD and OCD symptoms improvement.

Regarding our path analysis model, in the first instance, it can be hypothesized that bodily signals may be highly relevant in identifying emotions (i.e., disgust, guilt, and shame investigated in our study). In fact, the importance of physiological modifications in the development of one’s own emotions is highlighted by emotion theories, along with the wide overlap between brain areas involved in somatosensory processing and the sense of emotional strength. For at least four decades, the role of the ability to detect physiological modifications and how this affects how intense an emotion is perceived has been investigated (120). In fact, it has been proposed that disgust may represent an embodiment of moral judgment in terms of gut feelings (121), that psychological disgust can be disrupted by an antiemetic, and that doing so has consequences for moral judgments (122). In addition, it has been proposed that subjective body weight may represent an embodiment of guilt, and in particular, compared to activities that required less physical effort, an induction of guilt had an impact on the perceived amount of struggle required to finalize physical tasks (123). Furthermore, guilt was associated with alterations in gastric rhythms, electrodermal activity, and swallowing rate (124). Finally, shame showed distinct psychophysiological responses. Participants who were experiencing shame were found to raise their nasal temperature (125).

Post-traumatic guilt and shame were both cross-sectionally linked to the intensity of PTSD symptoms, and it has long been recognized that emotions of guilt and shame were related to the number of traumatic event categories that participants had experienced (126). However, recently, 41 women who suffered from sexual trauma were investigated, and it has been shown that trauma-related shame and guilt were found to act as prospective predictors of PTSD symptoms (23). Analogously, it has been demonstrated that disgust is an effective predictor of MC in PTSD (43) and OCD (127). In particular, more recent research has shown that sexual disgust is a specific affective predictor of MC (44). In turn, both TDDS and VOCI-MC are able to predict OCD scores (128). Specifically, regarding OCD symptoms, our results are in line with the literature. In fact, MC was a partial mediator of disgust propensity effects in triggering contamination fear (127, 129), and, in particular, of contamination fear based upon disgust avoidance (45). Regarding PTSD symptoms, it was shown that the tendency to engage in avoidance coping positively mediated relations between baseline MC and daily PTSD symptoms, and baseline PTSD symptoms and daily MC. Furthermore, daily avoidance coping positively mediated associations between daily MC and subsequent daily PTSD symptoms (130). Overall, these results support a mutual maintenance model of PTSD symptoms and trauma-related MC mediated by avoidance coping. Conversely, in our study, MC was not found to be related to IES-R. As already specified in the aforementioned case of disgust, our finding could be related to the fact that in our study, we simultaneously assessed and analyzed variables related to both PTSD and OCD. Therefore, when PTSD and OCD symptoms are assessed together, MC may preferentially serve as a mediator of disgust and a maintenance factor for OCD symptoms in order to manage the underlying trauma and PTSD. Unfortunately, the COVID-19 pandemic has been considered both a new traumatic trigger for PTSD (1, 2) and dissociative disorders (55) and a potential trigger, or reinforcement, of OCD (131, 132). A population mental health perspective informed by clinical psychology, psychiatry, and dissemination and implementation science related to effective treatments is ideally suited to address the broad, multi-faceted, and long-lasting mental health impact of the pandemic (133). Our results highlight evidence suggesting that EMDR may be considered an effective intervention therapy for the immediate and long-term psychological effects of the COVID-19 pandemic (55). Taken together, our findings suggest that for people suffering from PTSD, which may have also been triggered by COVID-19 quarantine and isolation, targeting guilt- and shame-related memories and images with EMDR may exert beneficial effects and promote the mitigation of PTSD-related symptoms. Targeting disgust-related memories and images may also be beneficial in order to reduce OCD symptoms (e.g., contamination-related symptoms) in cases where significant obsessive-compulsive symptoms are also present. In addition, as highlighted by path analysis, bodily sensations may generate subsequent emotions and dysfunctional cognitions and symptoms. Therefore, during EMDR intervention, it may be particularly important to focus on the body points that generate disturbing sensations, supporting the patient in translating into words what the bodily sensations seem to evoke.

To the best of our knowledge, this is the first time that ML methods have been applied to EMDR and related psychological domains, in terms of diagnostic prediction after EMDR treatment in a randomized controlled trial. ML models have shown promising advantages in solving classification problems. The issue we intended to address using ML models was the following: which predictors best distinguish between participants who underwent EMDR treatment and controls? We found that levels of BPQ-22, GASP, TDDS, VOCI-MC, DOCS, and IES-R after EMDR treatment as predictors were able to correctly classify participants who underwent EMDR treatment from controls. The most efficient rules were obtained by Naïve Bayes and Random Tree algorithms, which correctly classified the subjects in the two groups. Overall, both algorithms yielded an overall accuracy of 98.08% (Naïve Bayes, no. correct classification: 51/52, accuracy: 98.18%, area under ROC curve: 0.954; Random Tree, no. correct classification: 51/52, accuracy: 98.08%, area under ROC curve: 0.949).

Nevertheless, the following limitations should be taken into account when interpreting our results: a) a relatively small sample was used in our research, and larger samples should be used in future research; b) the generalizability of our findings may have been limited since subjects were self-selected; c) associations among variables may have been inflated since data are self-reported; d) not all of the measures we employed in our study have an already published validated Italian version since the paper is undergoing the review process; e) our results could be replicated using different measures. For example, we used a dimensional OCD symptom measure in our study, the DOCS (39, 73); future research could replicate our results using other categorical OCD symptom measures.

Notwithstanding these limitations, our research revealed that an EMDR intervention ameliorated BPQ-22, GASP, TDDS, VOCI-MC, DOCS, and IES-R scales in a sample of adult individuals with respect to a control sample. Our results promote the use of EMDR in individuals with PTSD and OCD symptoms related to the COVID-19 pandemic in order to prevent the persistence and maintenance of the symptoms. Notably, after acute stressful events, EMDR therapy may be a useful treatment for early intervention and long-term prevention of the development of psychological disturbances (134). EMDR intervention could be a useful therapy to foster integration in both clinical (135) and non-clinical populations (136).

## Data availability statement

The raw data supporting the conclusions of this article will be made available by the authors, without undue reservation.

## Ethics statement

The studies involving humans were approved by Ethics Committee, University of Pisa. The studies were conducted in accordance with the local legislation and institutional requirements. The participants provided their written informed consent to participate in this study.

## Author contributions

MM: Writing – review & editing, Supervision, Resources, Project administration, Methodology, Investigation, Data curation. AP: Writing – review & editing, Writing – original draft, Validation, Software, Resources, Methodology, Investigation, Formal analysis, Conceptualization.
