# Supplementary material for: Randomized trial on the effects of an EMDR intervention on traumatic and obsessive symptoms during the COVID-19 quarantine: a psychometric study
Source: Front Psychiatry. 2024 Jun 26;15:1369216. doi: 10.3389/fpsyt.2024.1369216 (PMC11233768; doi:10.3389/fpsyt.2024.1369216)
Supplement: Supplementary file 1 [file Table_1.docx]

*Supplementary Table 1*

*Overview of the EMDR protocol during the eight-week treatment.*

| **Session** | **EMDR-related activities** |
| --- | --- |
| **1** | Anamnesis; presentation of the intervention; invitation to fill in the psychometric tools for the assessment |
| **2** | Psychoeducation about trauma; Quality of life monitoring; Exercise on “Safe place” installation |
| **3** | Stabilization exercises. Training on the relational and mastery skills |
| **4** | Recent events protocol; Identification of traumatic scenarios and images and desensitizing first Points of Disturbance (PoD) with bilateral stimulation |
| **5** | PoDs identification and desensitization with bilateral stimulation |
| **6** | PoDs identification and desensitization with bilateral stimulation |
| **7** | PoDs identification and desensitization with bilateral stimulation |
| **8** | Review and closure |

*Supplementary Table 2*

*Group comparisons among the study measures between EMDR (n = 52) and Control (55) samples assessed pre-intervention and post-intervention.*

| Variable | Control - Pre | EMDR - Pre | *p* | Control - Post | EMDR - Post | *p* |
| --- | --- | --- | --- | --- | --- | --- |
| 1. DASS-21 | 18.78 (13.28) | 17.42 (13.24) | = .803 | 19.49 (13.09) | 9.29 (4.59) | < 0.001 |
|  | 14 [9] | 15 [19] |  | 18 [20.75] | 9 [8] |  |

Note: *p* = *p*-value resulting from Conover’s post-hoc test from Two-way Analysis of Variance on Ranks for row 1. DASS-21 - Depression Anxiety Stress Scale – 21. Mean and standard deviation (in brackets), and median and interquartile range (in square brackets) are shown for row 1.

*Supplementary Table 3*

*Group comparisons among the study measures within EMDR (n = 52) and Control (55) samples assessed pre-intervention and post-intervention.*

| Variable | Control - Pre | Control - Post | *p* | EMDR - Pre | EMDR - Post | *p* |
| --- | --- | --- | --- | --- | --- | --- |
| 1. DASS-21 | 18.78 (13.28) | 19.49 (13.09) | = 0.638 | 17.42 (13.24) | 9.29 (4.59) | < 0.001 |
|  | 14 [9] | 18 [20.75] |  | 15 [19] | 9 [8] |  |

Note: *p* = *p*-value resulting from Conover’s post-hoc test from Two-way Analysis of Variance on Ranks for row 1. DASS-21 - Depression Anxiety Stress Scale – 21. Mean and standard deviation (in brackets), and median and interquartile range (in square brackets) are shown for row 1.

*Supplementary Table 4*

*Hierarchical regression analyses predicting DOCS total score and IES-R total score from the other interval scales for group EMDR sample post-intervention (n = 52).*

| Predictor | β | *t* | *p* |
| --- | --- | --- | --- |
| Criterion: DOCS-TOT | | |  |
| *Model 1* - *Adjusted R*^2^ = -.016 | | |  |
| DASS-21 | -.060 (.065) | -.424 | .674 |
|  |  |  |  |
| *Model 1* - *Adjusted R*^2^ = .042 | | | |
| DASS-21 | -.098 (.079) | -.579 | .566 |
| BPQ-22 | .128 (.191) | .714 | .479 |
| GASP | -.004 (.107) | -.028 | .978 |
| TDDS | -.007 (.058) | -.046 | .964 |
| VOCI-MC | .383 (.414) | 2.299 | .026* |
| IES-R | .112 (.151) | .767 | .447 |
|  | | |  |
| Criterion: IES-R-TOT | | |  |
| *Model 1* - *Adjusted R^2^* = -.019 | | |  |
| DASS-21 | -.027 (.063) | -.192 | .849 |
|  |  |  |  |
| *Model 2* - *Adjusted R^2^* = .045 | | | |
| DASS-21 | -.151 (.077) | -.880 | .383 |
| BPQ-22 | .259 (.184) | 1.446 | .155 |
| GASP | -.159 (.104) | -1.020 | .313 |
| TDDS | -.058 (.057) | -.402 | .690 |
| VOCI-MC | .367 (.410) | 2.151 | .037* |
| DOCS | .115 (.146) | .767 | .447 |

Note: 1. BPQ-22 - Body Perception Questionnaire - 22; 2. GASP - Guilt and Shame Proneness scale; 3. TDDS - Three Domain of Disgust Scale; 4. VOCI-MC - Vancouver Obsessional Compulsive Inventory – Mental Contamination; 5. DOCS - Dimensional Obsessive Compulsive Scale; 6. IES-R - Impact of Event Scale – Revised; 7. DASS-21 - Depression Anxiety Stress Scale – 21. * *p* < 0.05, ** *p* < 0.01, *** *p* < 0.001
